# Ultrasound-Assisted Activated Carbon Adsorption Au(CN)_2_^−^

**DOI:** 10.3390/ma18071526

**Published:** 2025-03-28

**Authors:** Yunlong Bai, Hui Ge, Qi Li, Feng Xie, Wei Wang

**Affiliations:** 1 Key Laboratory for Ecological Metallurgy of Multimetallic Ores (Ministry of Education), Northeastern University, Shenyang 110819, China; baiyl@smm.neu.edu.cn (Y.B.); wangwei@smm.neu.edu.cn (W.W.); 2 School of Metallurgy, Northeastern University, Shenyang 110819, China; 3 Key Laboratory for Recycling of Nonferrous Metal Resources, Northeastern University, Shenyang 110819, China; 4 BYD Automobile Industry Co., Ltd., Shenzhen 518000, China; liq_neu@163.com

**Keywords:** ultrasound, gold adsorption, activated carbon

## Abstract

Ultrasound is introduced into the gold adsorption process by activated carbon to improve the adsorption efficiency. The effects of ultrasonic equipment, initial pH value, amount of activated carbon, initial CN^−^ concentration, and temperature on the Au(CN${)}_{2}^{-}$ adsorption onto activated carbon were investigated. The adsorption results show that the water bath ultrasonic generator is more beneficial to promote the gold adsorption onto activated carbon than the probe ultrasonic equipment. With the introduction of ultrasound, the gold adsorption capacity of activated carbon is obviously improved, and the gold balance loading is increased by about 37%. The Freundlich model can describe well the adsorption behavior of Au(CN${)}_{2}^{-}$ onto activated carbon with or without ultrasound, which indicates that the Au(CN${)}_{2}^{-}$ adsorption onto activated carbon belongs to multi-molecular chemisorption. Compared with the pseudo-first-order equation and intra-particle diffusion equation, the pseudo-second-order equation can better fit dynamic data. Through X-ray diffraction (XRD), X-ray photoelectron spectroscopy (XPS), infrared spectroscopy (FT-IR), BET specific surface area, SEM, and particle size distribution analysis, it is proved that the enhancement of the gold adsorption onto activated carbon by ultrasound is due to the continuous action of ultrasound, which reduces the particle size and ultrasonication increases specific surface area through physical fragmentation effects and improves active site accessibility by reducing mass transfer resistance of activated carbon, rather than changing the adsorption mechanism of gold onto activated carbon.

## 1. Introduction

The cyanidation technique utilizes common alkaline metal cyanides, including sodium cyanide and potassium cyanide, to leach gold and silver from minerals via electro-oxidation mechanisms. The reaction produces metal–cyanide complexes (Equation (1)) that facilitate selective extraction from mineral substrates. The advantages of cyanidation leaching include high efficiency, low cost, and strong adaptability to minerals. At present, about 90% of gold mines in the world are leached by cyanidation [1]. Although cyanides are highly toxic, after more than a century of development, a mature industrial process and a relatively complete theoretical system have been established, so it is still difficult to replace cyanidation leaching in a short time with non-cyanide leaching.

(1) $${4\mathrm{Au}+8\mathrm{CN}}^{-}{+O}_{2}{+2H}_{2}O\to {4\mathrm{Au}(\mathrm{CN})}_{2}^{-}{+4\mathrm{OH}}^{-}$$

After cyanidation leaching, gold recovery from ore pulp or cyanide can be accomplished through various techniques, including activated carbon adsorption [2], zinc cementation [3], and ion exchange [4]. With inherent advantages such as high efficiency, low cost, and simple operation, activated carbon adsorption has currently become the most widely used method for gold recovery in industries. Based on the different stages of active carbon addition, it can be divided into two adsorption methods: carbon in pulp (CIP) and carbon in leach (CIL). Additionally, for optimized sieve-based separation of gold-bearing activated carbon from the ore pulp, the activated carbon used for adsorption is usually large particles of coconut-shell active carbon with a particle size of about 1.0–3.0 mm. However, due to the increase in particle size, the gold adsorption effect of activated carbon decreases significantly with the increase in particle size.

To address the demands of rapid and efficient industrial development, researchers synthesize materials with large specific surface area and modify traditional activated carbon to improve the adsorption and recovery efficiency of gold [5]. Currently, some new adsorbents such as graphene oxide–polyethylenimine hydrogel [6], zirconium-based metal organic frameworks [7], and trimesoyl chloride-crosslinked polyethyleneimine [8] were synthesized to adsorb gold. In addition, traditional activated carbon can be modified by thiourea thermal decomposition [9], sulfur impregnation [10], and magnetic [1] methods to improve its adsorption capacity for gold. Although these new adsorbents have obvious advantages over traditional coconut-shell activated carbon in terms of gold adsorption capacity, their expensive price, complicated synthesis steps, and poor wear resistance limit their industrial application.

In recent years, ultrasound has been proven to be able to enhance the adsorption efficiency. Zhang et al. [11] observed that introducing ultrasound to the adsorption of Congo red by Mg-Al-CO_3_ layered double hydroxide could significantly shorten the adsorption equilibrium time compared to stirring and oscillation. Daware and Gogate [12] compared the conventional and the ultrasound-assisted adsorption effect of tea waste on pyridine wastewater, and the results showed that ultrasound-assisted adsorption could significantly improve the adsorption degree and accelerate the adsorption rate. In addition, ultrasound also exerts a positive effect on the adsorption process when activated carbon is used as an adsorbent. Jing et al. [13] observed that introducing ultrasound could improve the adsorption rate in the initial stage when activated carbon was used to adsorb Cr(VI). Although ultrasound can enhance the adsorption process of some adsorbents to adsorbates, empirical data on the ultrasound-assisted adsorption effect of activated carbon on gold remain scarce.

This work systematically compared the adsorption effect of gold onto activated carbon under the traditional stirring process versus the ultrasound-assisted process. The influences of ultrasonic equipment, initial pH, activated carbon dosage, and CN^−^ concentration on gold adsorption were investigated in detail. The adsorption kinetics and isotherms were studied, and different models were used to study the adsorption behavior of gold on activated carbon. Finally, the enhancement mechanism of activated carbon adsorption gold by ultrasound was described.

## 2. Experimental

### 2.1. Materials

All experimental solutions were meticulously prepared using deionized water. Analytical reagents including silver nitrate, p-dimethylamino benzyl rhodanine, acetone, potassium chloride, hydrochloric acid, sodium cyanide, activated carbon, and sodium hydroxide. (Sinopharm Chemical Reagent Co., Ltd., Shanghai, China) The purity of the gold powder used in the experiment was 99.99% (Shenyang dongchuang precious metal material Co., Ltd., Shenyang, China) Activated carbon was activated carbon with particle size of 1180~1700 μm was selected by manual sieving and washed until the washing liquid was clear and transparent, and then dried at 105 °C for 24 h.

### 2.2. Preparation of Au(CN${)}_{2}^{-}$

The Au(CN${)}_{2}^{-}$ solution was synthesized via dissolving gold dust in sodium cyanide solution. The gold concentration, free cyanide concentration, and pH of the solution was adjusted by changing the added amount of gold dust, sodium cyanide, and sodium hydroxide.

### 2.3. Adsorption Experiments

The point of zero charge (pH_PZC_) of activated carbon was determined by the pH drift method, as follows: 0.2 g of activated carbon was added to a series of 100 mL 0.1 mol/L KCl solutions, and the initial pH of the solution was adjusted to 2.00, 3.03, 4.03, 5.05, 5.98, 6.98, 8.17, 9.12, 9.91, 11.05, and 12.00 by adding 1.0 mol/L HCl or NaOH solution. After being placed in a shaker for 24 h, the pH of the supernatant was measured by a pH meter. The pH_PZC_ value of the activated carbon is identified at the intersection point where the initial pH curve meets the final pH curve.

The amount of gold adsorbed on activated carbon can be calculated according to Equation (2):

(2) $$q=\frac{C_{0}V_{0}-C_{t}V_{t}}{m}$$

where *C*_0_ is the initial gold concentration (mg/L), *V*_0_ is the initial volume (L) of gold-containing solution, *C*_t_ is gold concentration (mg/L) at time t (h), *V*_t_ is the volume at time t, and *m* is the weight of the activated carbon (g).

**Figure 1:**
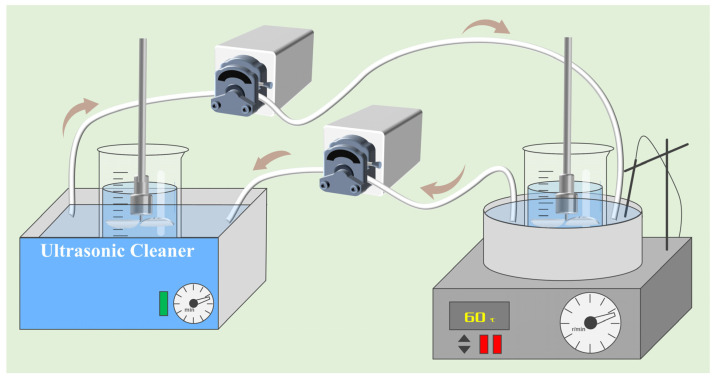
Experimental setup for ultrasound-assisted and conventional adsorption.

For the static adsorption experiments, 150 mL of a certain concentration of gold standard solution was placed in a 200 mL beaker, and a certain amount of activated carbon added. For conventional adsorption, the beaker was positioned within a thermostatic water bath; for ultrasonic-assisted adsorption, the beaker was placed in an ultrasonic cleaner, as shown in Figure 1. Mechanical stirring, operating at 200 r/min, was uniformly applied to both conventional and ultrasound-assisted adsorption. The adsorption solution was subjected to filtration for activated carbon removal when the adsorption was completed, after which the filtrate was analyzed for the pH value, free cyanide concentration, and gold concentration using standardized analytical protocols.

A series of gold standard solutions with concentrations of 10.22, 25.5, 45.78, 77.72, 102.58, 169.74, 225.29, and 250.10 mg/L were prepared for the adsorption thermodynamic experiments. A 200 mL beaker containing 150 mL of gold standard solution with predetermined concentration was charged with 0.2 g of activated carbon. Adsorption processes were conducted at three distinct thermal conditions (298 K, 313 K, 328 K) over an 8-h period.

A series of 200 mL beakers were loaded with 150 mL portions of 50 mg/L gold standard solution, and 0.2 g of activated carbon was added and adsorbed for different times at a temperature of 298 K to conduct the adsorption kinetics experiments.

### 2.4. Characterization of Samples

The solution pH was measured using a pH meter, PB-10 (Sartorius, Götting Germany). Atomic absorption spectrometry analysis, AAS, Hitachi Z-2300 (Hitachi, Tokyo, Japan) was employed to determine gold content in the solution samples. The free cyanide concentration was analyzed by silver nitrate titration. FT-IR (Nicolet 380, Thermo Corporation, Waltham, MA, USA), scanning electron microscopy (SEM, Quanta 250FEG, Thermo Fisher Scientific, Hillsboro, OR, USA), and X-ray photoelectron spectroscopy (XPS, EscaLab 250Xi, Thermo Fisher Scientific, Waltham, MA, USA) were applied to analyze the physical and chemical properties of the activated carbon. Phase composition analysis of solid samples was conducted via XRD (Bruker D8 advance, BRUKER, Karlsruhe, Germany) employing Cu Kα radiation with a scanning duration of 15 min over an angular range of 10° to 90°. The pore structure of the samples was characterized by an automated gas adsorption analyzer (Micromeritics ASAP 2020, Micromeritics Instrument Corporation, Norcross, GA, USA).

## 3. Results and Discussion

### 3.1. Effect of Ultrasonic Device

The effect of the ultrasonic device on the adsorption of Au(CN${)}_{2}^{-}$ by activated carbon was investigated. As shown in Table 1, after adsorption for the same time, the adsorption amount of activated carbon assisted by the water-bath-type ultrasonic device was higher than that for the probe-type ultrasonic device. Therefore, the water-bath-type ultrasonic generator was employed to coordinate the adsorption of gold by activated carbon in the subsequent adsorption experiments. The water-bath ultrasonic generator performed better in promoting the adsorption of Au(CN${)}_{2}^{-}$ on activated carbon, mainly due to its uniform energy distribution and stable activation effect. This enabled the bath ultrasonic waves to enhance the adsorption performance of the activated carbon in a more comprehensive way, thus becoming a more effective ultrasonic aid in this study.

### 3.2. Effect of Initial pH

**Figure 2:**
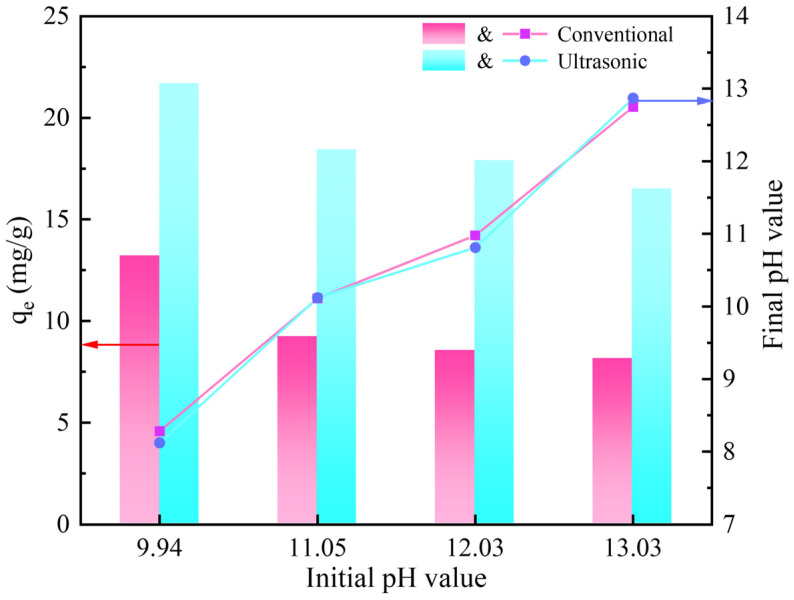
Effect of initial pH on the gold adsorption.

**Figure 3:**
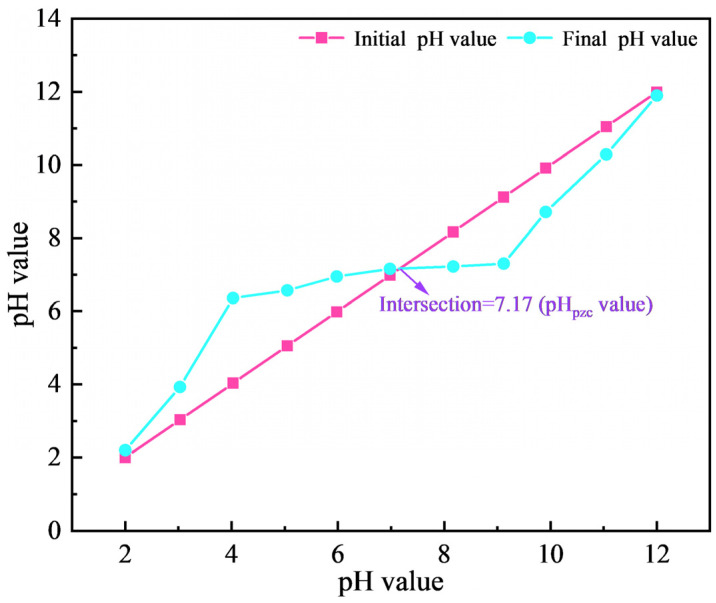
Point of zero charge (pH_PZC_) of the activated carbon determined by the pH drift method.

The solution pH is one of the important factors affecting the adsorption process, so the effect of the initial solution pH on the gold adsorption was studied. Since more than 50% of CN^−^ exists in the form of HCN when the pH is lower than 9, and the actual cyanide leaching of gold is usually carried out at a pH above 10, the experiments in this section were carried out in the pH range of 9.94–13.30. As shown in Figure 2, the adsorption of gold on the activated carbon decreased gradually with the increase in pH, whether with and without ultrasound. In addition, the adsorption capacity of the ultrasound-assisted adsorption was significantly better than that of the conventional adsorption under different pH conditions. Among them, the adsorption of gold reached a maximum value of 21.7 mg/g and 13.2 mg/g at an initial pH of 9.94 in both the presence and absence of ultrasonic waves, respectively. At the same time, after the adsorption under different initial pH, the solution pH value was reduced and the pH change trend in the solution was similar, whether with or without ultrasound. The point of zero charge (pH_PZC_) is a significant determinant affecting the surface charge of the adsorbent. As shown in Figure 3, the pH_PZC_ (measured with activated carbon “as is”) of the activated carbon was determined by the pH drift method to be 7.17. When the solution pH is higher than 7.17, the activated carbon is easy to deprotonate, resulting in its surface being negatively charged and electrostatically repulsive to Au(CN${)}_{2}^{-}$ ions, and the pH of the solution gradually decreases. Accordingly, the higher the pH of the solution, the less favorable it is for the adsorption of Au(CN${)}_{2}^{-}$ [14,15]. Taking into account the safety and adsorption effect, the pH of the solution was fixed to 9.9 in the subsequent adsorption experiments.

### 3.3. Effect of Activated Carbon Dosage

**Figure 4:**
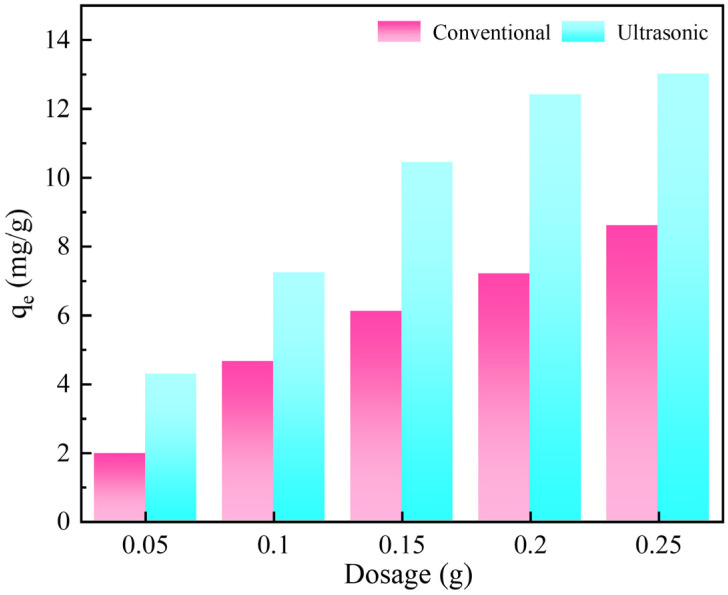
Effect of activated carbon dosage on the gold adsorption.

The effect of activated carbon dosage on gold adsorption is shown in Figure 4. Regardless of the presence or absence of ultrasound, the gold adsorption by the activated carbon increases gradually with the increase in activated carbon dosage. This is attributed to the limited saturation adsorption capacity of activated carbon; increasing activated carbon dose can lead to a larger specific surface area and more available adsorption sites. Using different dosages of activated carbon, the adsorption amount of activated carbon to gold under the ultrasonic action is higher than that of conventional adsorption, indicating that ultrasound obviously promotes gold adsorption. Due to the adsorption amount of 0.25 g, the activated carbon was not significantly higher than that of 0.2 g under the ultrasonic action; thus, 0.2 g activated carbon was used for subsequent adsorption experiments.

### 3.4. Effect of CN^−^ Concentration

**Figure 5:**
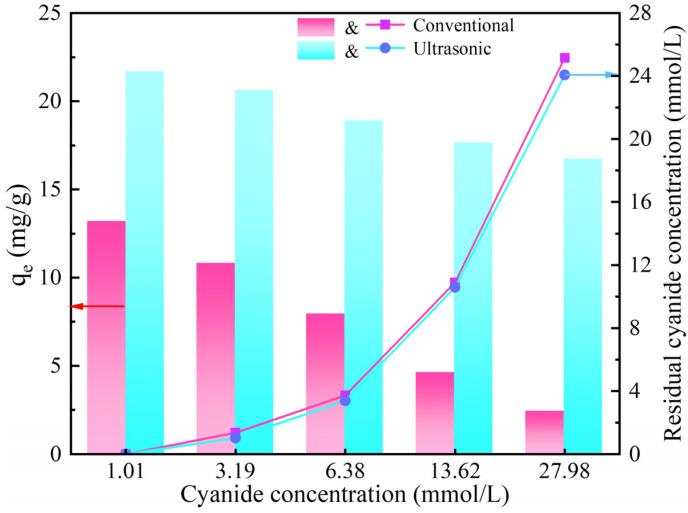
Effect of initial CN^−^ concentration on the gold adsorption.

Ion strength usually influences adsorption, as the different ions may compete for adsorption sites on the adsorbent. Therefore, the effect of CN^−^ concentration on gold adsorption by activated carbon was investigated and the experimental outcomes are presented in Figure 5. With the increase in CN^−^ concentration in the solution, the adsorption capacity of the activated carbon for gold gradually decreased, and the decreasing trend was more obvious in the conventional adsorption. In addition, the adsorption capacity of the activated carbon for gold under ultrasonic action was obviously higher than that of conventional adsorption, and the difference was more obvious with the increase in CN^−^ concentration. By monitoring the CN^−^ concentration changes in the solution before and after adsorption, it was found that the CN^−^ concentration obviously decreased after adsorption. Furthermore, CN^−^ concentration was slightly lower than that of conventional adsorption under ultrasonic action, indicating that CN^−^ could form competitive adsorption with Au(CN${)}_{2}^{-}$, which was also confirmed in the literature [16,17]. Considering that the adsorption capacity of the activated carbon for gold and CN^−^ was improved by ultrasound, the ultrasonic cavitation effect enhances the adsorption performance through the following pathways:

(a) Physical fragmentation: The microjet generated by the collapse of high-frequency cavitation bubbles can strip the inert layer on the surface of the activated carbon, exposing more potential adsorption sites.

(b) Pore activation effect: Ultrasonic energy promotes the desorption of impurities within the adsorbent pores, expanding the effective adsorption space (increase in BET specific surface area).

To create activated carbon with a strong gold adsorption capacity, the CN^−^ concentration was fixed at 1 mmol/L in the subsequent adsorption experiments.

### 3.5. Adsorption Isotherm

**Figure 6:**
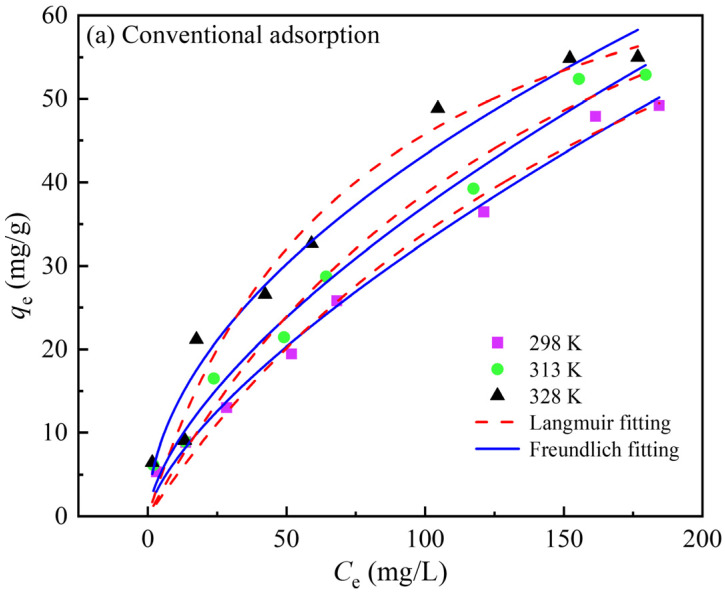

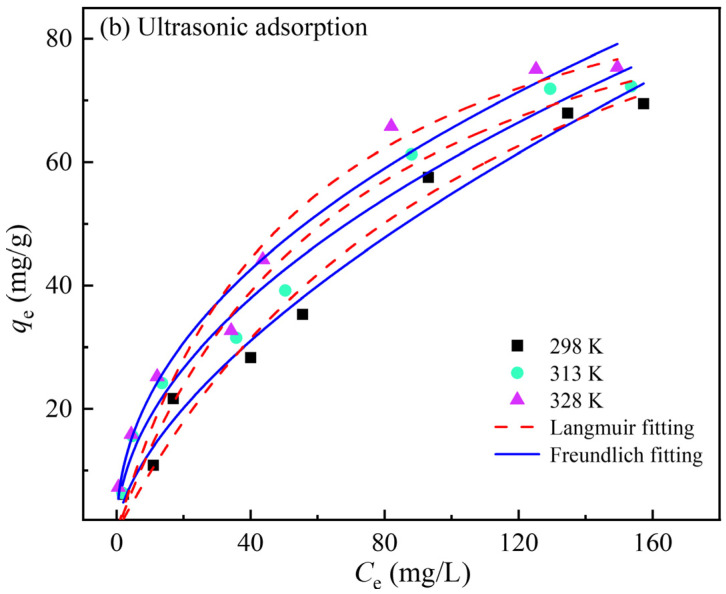
Adsorption isotherms of Au(CN${)}_{2}^{-}$ onto activated carbon at different temperatures in the absence (**a**) and presence (**b**) of ultrasound, and fitting by the Langmuir and Freundlich models.

The adsorption isotherm is an important curve to characterize the adsorption state. To further investigate the relationship between the equilibrium adsorption amount of the activated carbon and gold concentration in the solution, the adsorption experiment using gold standard solutions with different Au(CN${)}_{2}^{-}$ concentrations was carried out at 298, 313, and 328 K, and the isotherm data were described by Langmuir and Freundlich isotherm models. The Langmuir model, expressed as Equation (3), posits that the adsorbing molecules are adsorbed on the homogeneous surface of the adsorbent in a single molecular layer, and there is no interaction between the adsorbing substances. The Freundlich model, which can be expressed as Equation (4), provides a suitable framework for the adsorption of adsorbates on the non-homogeneous surface of the adsorbent, where adsorption capacity is significantly influenced by the chemical properties of the adsorbent surface and the interaction between the adsorbates [7,18]. By fitting the adsorption data and calculating the isotherm parameters by two models, the results are shown in Figure 6 and Table 2 and Table 3. The linear correlation coefficient R^2^ value for the Freundlich model is higher than that for the Langmuir model, whether with or without ultrasound, and the calculated adsorption amount is very close to the experimental data. Therefore, the Freundlich model is more suitable than the Langmuir model to describe the adsorption behavior of Au(CN${)}_{2}^{-}$ on activated carbon, indicating that the adsorption is not a single-molecule physical adsorption, but a multi-molecule chemical adsorption. In addition, the saturation adsorption amount of gold on activated carbon increases under progressively higher temperature conditions, and the adsorption capacity of the activated carbon for Au(CN${)}_{2}^{-}$ could be obviously improved by ultrasound, and the equilibrium loading amount of gold was increased by about 37% under the ultrasonic action.

(3) $$q_{e}=q_{m}\frac{k_{L}C_{e}}{1+C_{e}}$$

(4) $$q_{e}=k_{F}C_{e}^{\frac{1}{n}}$$

where *C*_e_ is gold concentration in solution at adsorption equilibrium (mg/L), *q*_e_ and *q*_m_ are the adsorption capacity at equilibrium and the maximum adsorption capacity (mg/L), respectively, *k*_L_ is the Langmuir model equilibrium constant (L/mg), *k*_F_ is the Freundlich model equilibrium constant ((mg/g)(L/mg)^1/n^), and *n* is a Freundlich constant.

**Figure 7:**
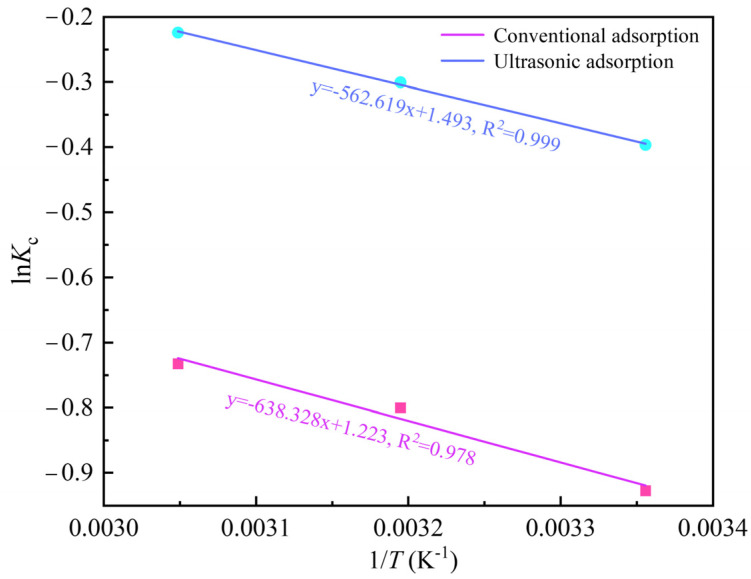
Van’t Hoff linear plots of ln*k*_c_ against 1/*T*.

For the purpose of describing the adsorption properties of activated carbon, the thermodynamic equation shown in Equations (5)–(8) was further used to analyze its thermodynamic parameters ∆*G*^o^, ∆*H*^o^, and ∆*S*^o^ [9,19]. As shown in Figure 7 and Table 4 and Table 5, it can be found that ∆*G*^o^ are all positive values, which is contrary to the conclusion of most researchers. Their research results show that ∆*G*^o^ is usually negative in the adsorption reaction process, indicating that the adsorption reaction is feasible and spontaneous [14,15,20]. However, from the calculation process of ∆*G*^o^ shown in Equations (5) and (6), if the adsorbent has sufficient adsorption capacity for the adsorbate, *K*_c_ will be greater than 1; thus, ∆*G*^o^ will be negative. On the contrary, if the adsorbent has limited adsorption capacity for the adsorbate, *K*_c_ will be less than 1, and ∆*G*^o^ will be positive. Therefore, whether the adsorption reaction can be spontaneous is not the only factor affecting the sign of ∆*G*^o^; however, the more negative the ∆*G*^o^ value, the stronger the adsorption capacity of the adsorbent for the adsorbate. For example, the calculation results show that ∆*G*^o^ is more negative under ultrasonic action than under conventional adsorption, indicating that the adsorption capacity of activated carbon for Au(CN${)}_{2}^{-}$ is enhanced by ultrasound, which is consistent with the actual adsorption experiment results. Similarly, the work of Sedira et al. [21] and Adebisi et al. [22] demonstrates that the positive ∆*G*^o^ reflects limited thermodynamic spontaneity of the adsorption process rather than its non-spontaneity. The adsorption of Au(CN) on activated carbon is a heat-absorption reaction, as evidenced by all the positive ∆*H*^o^ values, which aligns with experimental results, confirming that increasing the temperature favors the adsorption process. A positive ∆*S*^o^ is observed during the adsorption process of Au(CN${)}_{2}^{-}$ on activated carbon, indicating elevated degrees of freedom at the solid–liquid interface [23].

(5) $$K_{c}=\frac{q_{\mathrm{SPe}}}{C_{e}}$$

(6) $$△G^{0}=-RTlnK_{c}$$

(7) $$\Delta G^{0}=\Delta H^{0}-T\Delta S^{0}$$

(8) $$lnK_{c}=\frac{\left(\Delta S^{0}-\Delta H^{0}\right)}{RT}$$

where *K*_c_ is the thermodynamic equilibrium constant, *q*_SPe_ is the equilibrium concentration (mg/L) of gold adsorbed on the solid phase, *R* is the universal gas constant (8.314 J/mol/K), *T* is the adsorption process temperature (K), ∆*G*^o^ is Gibbs free energy (kJ/mol), ∆*H*^o^ is enthalpy (kJ/mol), and ∆*S*^o^ is entropy (J/mol/K).

The ultrasound-assisted adsorption capacity of coconut-shell activated carbon for Au(CN${)}_{2}^{-}$ is compared with data from the literature (Table 6). Of note is that compared with the synthesized adsorbent and modified activated carbon, the activated carbon adsorption capacity of Au(CN${)}_{2}^{-}$ is greatly improved via introducing ultrasound. Although its saturation loading amount is lower than that of sulfur-impregnated activated carbon, the ultrasound-assisted adsorption process is simple and does not require additional synthesis processing.

### 3.6. Adsorption Kinetics

**Figure 8:**
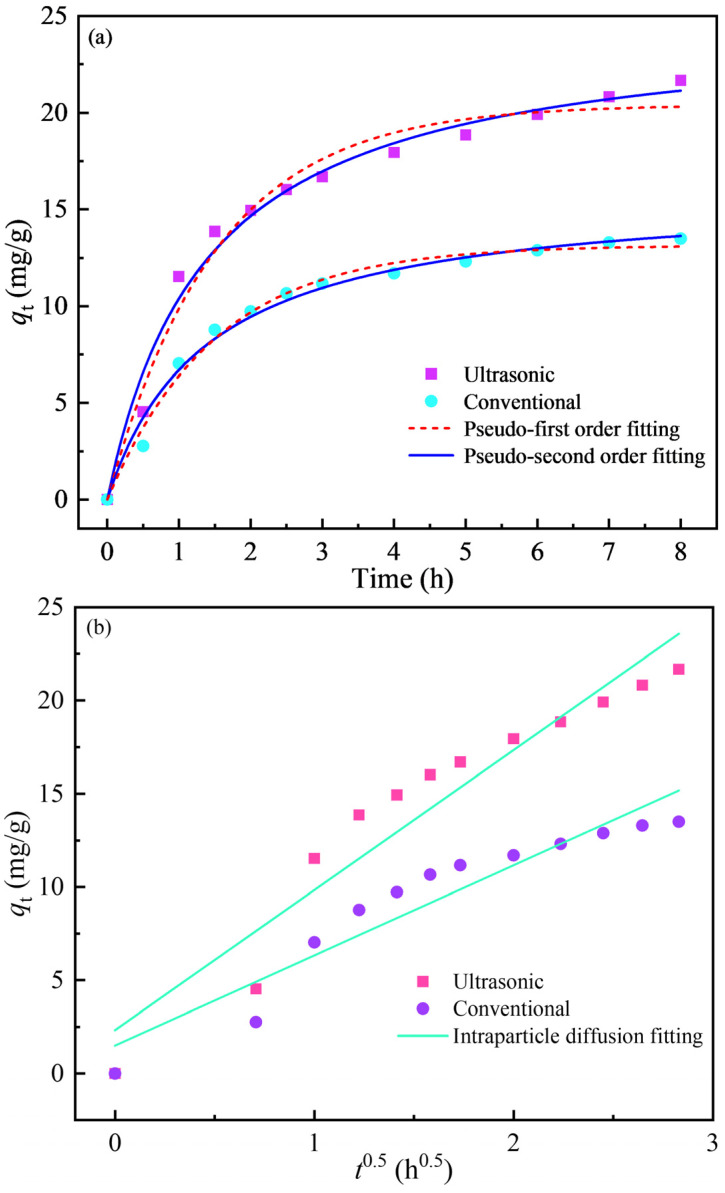
Adsorption kinetics of gold onto activated carbon, and fitting by the pseudo first-order, pseudo second-order (**a**), and intraparticle diffusion (**b**) models.

The adsorption kinetics of Au(CN${)}_{2}^{-}$ on the activated carbon is shown in Figure 8. The loading amount of gold on the activated carbon increases rapidly within the adsorption time of 0–3 h mol for both cases, with or without ultrasound. When the adsorption time exceeds 3 h, the increasing trend in gold loading amount slows. Compared with conventional adsorption, the gold adsorption capacity of the activated carbon increases more rapidly during the early phase of adsorption under the ultrasonic action, and the equilibrium adsorption amount increases by about 61%.

Adsorption kinetics can explain the adsorption mechanism [20]. With the aim of further understanding the difference in the adsorption kinetics of activated carbon for Au(CN${)}_{2}^{-}$ with and without ultrasound, pseudo first-order (Equation (9)), pseudo second-order (Equation (10)), and intraparticle diffusion (Equation (11)) models were used to simulate the adsorption kinetic data. Figure 8 and Table 7 and Table 8 present the fitting results and kinetic parameters [15]. The adsorption kinetics of Au(CN${)}_{2}^{-}$ on activated carbon are predicted with a certain deviation by the pseudo first-order model, which underestimates the adsorption amount in the gradual equilibrium stage. From the linear correlation coefficient R^2^ value, it is apparent that the adsorption kinetics of activated carbon for Au(CN${)}_{2}^{-}$ are closer to the pseudo second-order kinetic model for both situations, with or without ultrasound. This demonstrates that the adsorption of activated carbon for Au(CN${)}_{2}^{-}$ is dominated by chemical adsorption, and electron sharing and ion exchange may occur between the activated carbon and Au(CN${)}_{2}^{-}$ in this adsorption process [1,20]. Therefore, the better adsorption effect under the ultrasonic action can be attributed to the fact that the specific surface area of the activated carbon is increased by ultrasound, which makes the activated carbon possess more active sites per unit mass that can bind to Au(CN${)}_{2}^{-}$. For the intraparticle diffusion model, if the relationship between *q*_t_ and *t*_0.5_ passes through the origin, the rate-limiting step is governed by the intraparticle diffusion [15]. However, as shown in Figure 8b, the fitting results deviate from the origin, and the R^2^ values are only 0.92 and 0.90 under the presence and absence of ultrasound, respectively, indicating that the adsorption of Au(CN${)}_{2}^{-}$ on the activated carbon is not controlled by intraparticle diffusion.

(9) $$q_{t}=q_{e}\left[1-\exp\left(-k_{f}t\right)\right]$$

(10) $$q_{t}=\frac{q_{e}^{2}k_{s}t}{1+q_{e}k_{s}t}$$

(11) $$q_{t}=k_{i}t^{0.5}+C$$

where *q*_e_ and *q*_t_ (mg/g) are the gold adsorption amounts at equilibrium and time t (h), respectively; k_f_ (min^−1^), k_s_ (g/(mg·min)), and k_i_ (g/(mg·min^0.5^)) are the adsorption rate constants of the pseudo first-order, pseudo second-order adsorption, and intraparticle diffusion models, respectively; and *C* is the intercept (mg/g).

### 3.7. Mechanism of Ultrasonic Enhanced Adsorption

**Figure 9:**
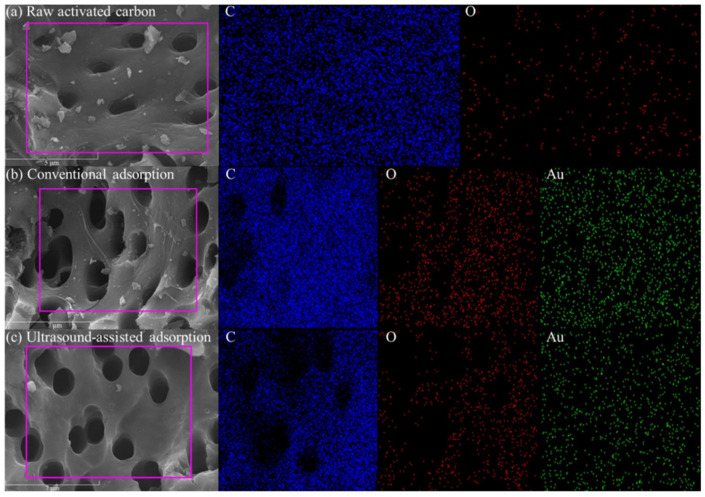
SEM images and main element distribution for raw activated carbon (**a**), activated carbon after conventional adsorption (**b**), and after ultrasound-assisted adsorption (**c**).

**Figure 10:**
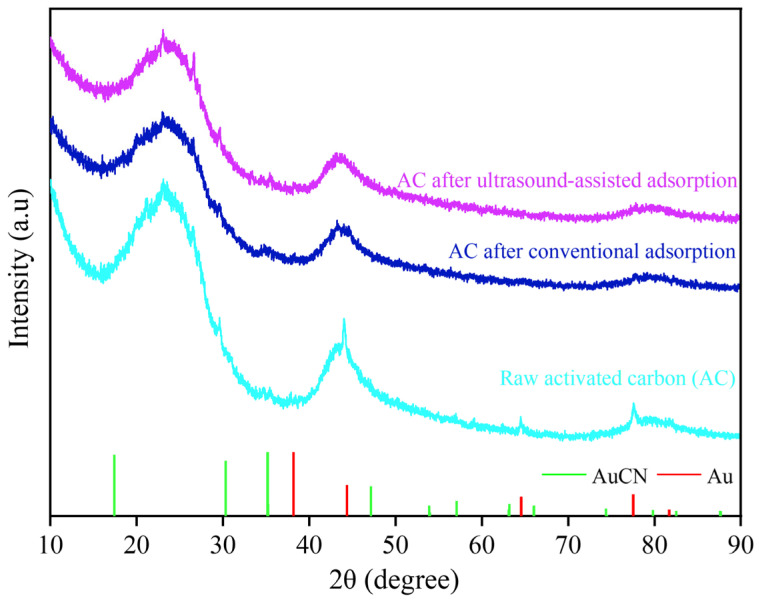
X-ray diffraction analysis of the activated carbon before and after adsorption.

**Figure 11:**
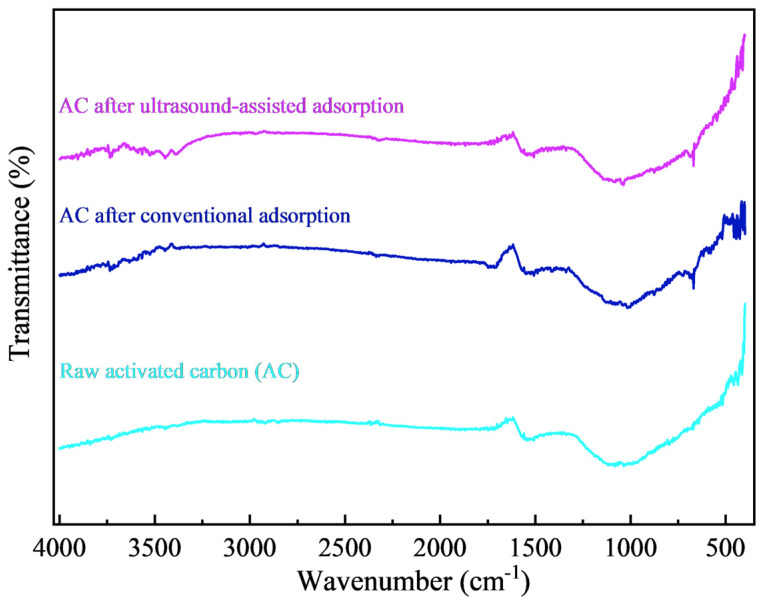
FT-IR spectra of the activated carbon before and after adsorption.

SEM images and the main element distribution of the activated carbon before and after adsorption are shown in Figure 9. There is no obvious difference in the surface morphology of the activated carbon before and after adsorption, which is an uneven and porous structure. The surface scanning results of the activated carbon before adsorption showed that its surface was mainly composed of C and O elements, where C is an inherent element of activated carbon, and O may come from metal oxides or non-metal oxides produced during the preparation of the activated carbon. The surface scanning results of the activated carbon after conventional and ultrasound-assisted adsorption showed that Au elements were evenly distributed, indicating that Au had been adsorbed on the activated carbon. The X-ray diffraction analysis of the activated carbon before and after adsorption is shown in Figure 10. The diffraction peaks of the activated carbon are broad and belong to amorphous carbon, which has not yet fully formed the crystal structure of a graphite layer. No obvious new diffraction peaks appeared after adsorption, indicating that the loading of Au(CN${)}_{2}^{-}$ on the activated carbon was below the XRD detection limit. The FT-IR spectra of the activated carbon before and after adsorption are shown in Figure 11. There is no obvious change in the infrared spectra of the activated carbon before and after adsorption, which potentially arises from the low content of Au(CN${)}_{2}^{-}$ loaded on the surface of the activated carbon.

The mechanism of Au(CN${)}_{2}^{-}$ adsorption by activated carbon has been widely studied since the introduction of the carbon slurry method, and the currently considered reasons why Au(CN${)}_{2}^{-}$ can be adsorbed by activated carbon are as follows [28]:

(1) Au(CN${)}_{2}^{-}$ is first adsorbed on the adsorbent and then reduced to metallic gold.

(2) Adsorption occurs by activated carbon as [M^n+^][Au(CN${)}_{2}^{-}$]_n_ ion pairs.

(3) Au(CN${)}_{2}^{-}$ is adsorbed on the surface of activated carbon due to electrostatic force, forming a double electric layer of cations and Au(CN${)}_{2}^{-}$ anions.

(4) Au(CN${)}_{2}^{-}$ is adsorbed on activated carbon by exchanging with OH^−^ ions, and then oxidized to water-insoluble AuCN.

(5) Au(CN${)}_{2}^{-}$ is adsorbed on the surface of activated carbon in the form of HAu(CN)_2_ or Au(CN${)}_{2}^{-}$-Na^+^ due to dispersion force.

Since the effects of initial pH, initial CN^−^ concentration, and temperature on conventional and ultrasound-assisted adsorption of gold had similar trends, and the SEM, XRD, and FT-IR detection results of the activated carbon after conventional and ultrasound-assisted adsorption were not significantly different, the mechanism of gold adsorption on the activated carbon was not significantly changed by ultrasound.

**Figure 12:**
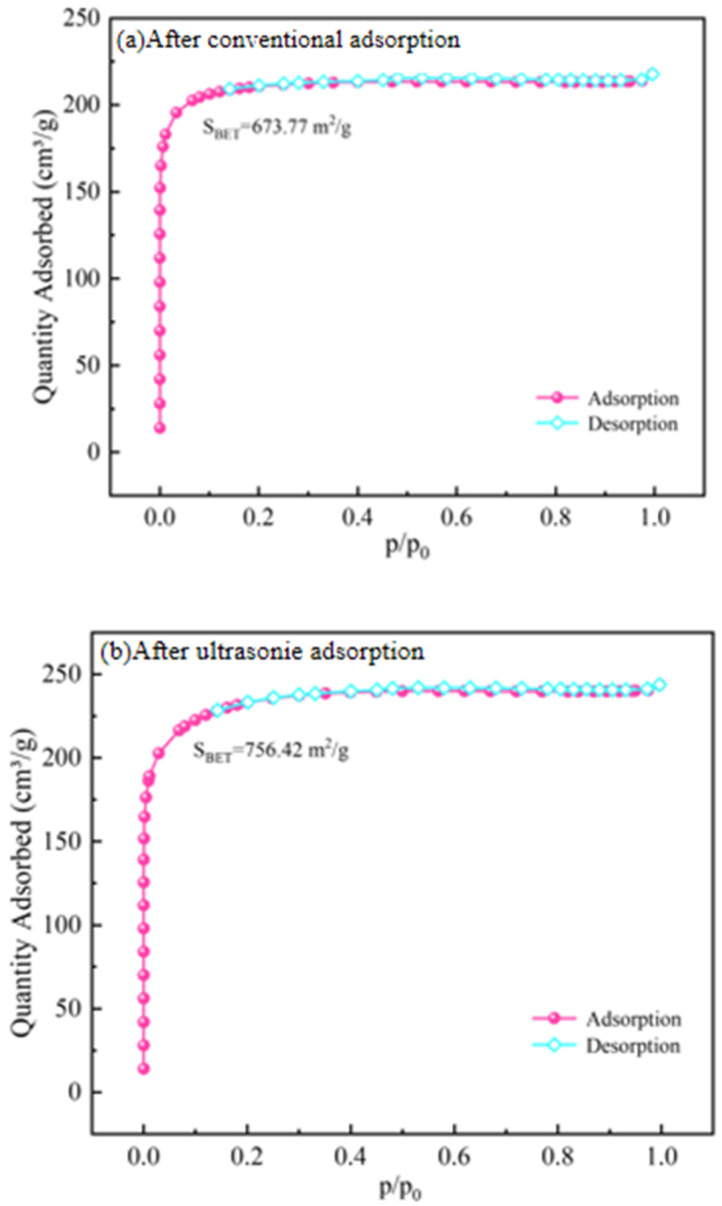
Adsorption–desorption isotherms of the activated carbon before and after adsorption.

The nitrogen adsorption–desorption technique was applied to analyze the BET specific surface area of the activated carbon before and after adsorption, and the results are shown in Figure 12. Following the classification system of adsorption isotherms by the International Union of Pure and Applied Chemistry (IUPAC), the nitrogen adsorption–desorption isotherm of the activated carbon belongs to type I, demonstrating that the coconut-shell activated carbon used is a microporous material. According to the nitrogen adsorption–desorption curve, the BET specific surface area, pore size, and pore volume of the activated carbon before and after adsorption were calculated. As shown in Table 9, the BET specific surface area of the activated carbon increased from 667.1 mg/g to 756.4 mg/g after ultrasound-assisted adsorption, while the BET specific surface area of the activated carbon remained basically unchanged after conventional adsorption. The particle size distribution of the activated carbon after conventional and ultrasound-assisted adsorption was analyzed, and the data are tabulated in Table 10. The initial particle size of the activated carbon was 1180–1700 μm, and about 12.8% of the activated carbon particles were less than 1180 μm after ultrasound-assisted adsorption, while only 4.5% of the activated carbon particles were less than 1180 μm after conventional adsorption. Based on these results, part of the activated carbon is refined under the action of ultrasonic waves, the specific surface area is increased, the surface diffusion and external mass transfer rate is accelerated, and the adsorption sites are increased, which leads to the enhanced adsorption capacity of activated carbon for Au(CN${)}_{2}^{-}$ under the ultrasonic action [29,30].

## 4. Conclusions

A water-bath ultrasonic generator can significantly improve the gold adsorption capacity of activated carbon. The adsorption of gold by activated carbon is an endothermic and entropy-increasing process, which is beneficial to the adsorption of Au(CN${)}_{2}^{-}$ by increasing temperature, reducing solution pH, and decreasing CN^−^ concentration. The experimental data of gold adsorption on activated carbon were fitted by isothermal models, and the Freundlich isothermal model was the most suitable for the gold adsorption process of activated carbon at different temperatures, indicating that the adsorption was mainly multi-molecular chemical adsorption. The pseudo second-order kinetic equation was the most suitable for the kinetic process of gold adsorption, indicating that the adsorption of Au(CN${)}_{2}^{-}$ on the activated carbon mainly occurred by ion exchange and chemical adsorption. Under the condition of temperature of 328 K, initial pH of 9.9, initial CN^−^ concentration of 1 mmol/L, and adsorbent dosage of 0.2 g, the strongest adsorption capacity of the activated carbon for gold was reached at 55.0 mg/g. However, introducing ultrasound could increase the gold loading amount to 75.4 mg/g. Ultrasound can reduce the particle size of activated carbon, increase the specific surface area, accelerate the mass transfer rate, and increase the adsorption sites, thus enhancing the adsorption capacity of activated carbon for Au(CN${)}_{2}^{-}$. The promotion of hydrogen bonds forming on the resin surface and increased surface roughness, both induced by ultrasound, were identified as critical factors facilitating the adsorption process through resin structural analysis.

## Figures and Tables

**Table 1:** Comparison of probe-type and water-bath ultrasonic devices in assisting activated carbon to adsorb gold.

Device	Power Output (W/cm^2^)	Frequency (kHz)	*q*_e_		
			Adsorption 2 h	Adsorption 4 h	Adsorption 6 h
Probe	80	20	2.88	4.25	5.91
Bath	80	20	3.56	5.58	7.23

**Table 2:** Fitting parameters of the Langmuir and Freundlich models for conventional adsorption at different temperatures.

T (K)	Langmuir Constants			Freundlich Constants		
	q_max_	B	R^2^	k	n	R^2^
298	108.373	0.005	0.985	1.353	1.443	0.993
313	100.431	0.006	0.974	1.939	1.560	0.985
328	80.249	0.013	0.967	3.944	1.923	0.968

**Table 3:** Fitting parameters of Langmuir and Freundlich models for ultrasound-assisted adsorption at different temperatures.

T (K)	Langmuir Constants			Freundlich Constants		
	q_max_	B	R^2^	k	n	R^2^
298	124.803	0.008	0.978	3.120	1.605	0.982
313	105.824	0.015	0.956	5.765	1.957	0.982
328	104.639	0.018	0.956	7.504	2.123	0.978

**Table 4:** *K*_c_ values at different temperatures.

Methods	298 K	313 K	328 K
Conventional	0.396	0.449	0.481
Ultrasound-assisted	0.673	0.740	0.799

**Table 5:** Thermodynamic parameters of Au(CN${)}_{2}^{-}$ adsorption onto activated carbon.

Methods	Temperature (K)	∆*G*^o^ (kJ.mol^−1^)	∆*H*^o^ (kJ.mol^−1^)	∆*S*^o^ (J mol^−1^.K^−1^)
Conventional	298	2.298	5.307	10.164
	313	2.082		
	328	1.997		
Ultrasound-assisted	298	0.982	4.678	12.415
	313	0.782		
	328	0.611		

**Table 6:** Comparison of the adsorption capacity of Au(CN${)}_{2}^{-}$ by the ultrasound-assisted coconut-shell activated carbon adsorption method with reported modified activated carbon and other adsorbents.

Intensification Methods or Other Adsorbents	Adsorption Capacity (mg/g)	Ref.
Sulfur-impregnated activated carbon	137.8	[10]
Magnetic-activated carbon	45.2	[1]
Burnt acid-washed crab-shell	10.0	[24]
Hydrotalcite-type anionic clay	60.0	[25]
Chitosan–polyacrylamide graft copolymers	10.2	[26]
Dowex 21 K XLT resin	32	[27]
Coconut-shell activated carbon	55.0	This work
Ultrasound-assisted activated carbon adsorption	75.4	This work

**Table 7:** Adsorption kinetic parameters obtained from the pseudo first-order, pseudo second-order, and intraparticle diffusion models under ultrasonic action.

Kinetic Models	Parameters	Value
Pseudo first-order model	q_e_ (mg/g)	20.42
	k_f_ (min^−1^)	0.661
	R^2^	0.979
Pseudo second-order model	q_e_ (mg/g)	24.82
	k_s_ (g/mmol/s)	0.029
	R^2^	0.984
Intraparticle diffusion model	k_i_ (mg/g/min^0.5^)	7.511
	C	2.329
	R^2^	0.918

**Table 8:** Adsorption kinetic parameters obtained from the pseudo first-order, pseudo second-order, and intraparticle diffusion models without ultrasonic action.

Kinetic Models	Parameters	Value
Pseudo first-order model	q_e_ (mg/g)	13.15
	k_f_ (min^−1^)	0.666
	R^2^	0.989
Pseudo second-order model	q_e_ (mg/g)	16.00
	k_s_ (g/mmol/s)	0.045
	R^2^	0.986
Intraparticle diffusion model	k_i_ (mg/g/min^0.5^)	4.837
	C	1.498
	R^2^	0.904

**Table 9:** BET surface area, total pore volume, and average pore size for the activated carbon before and after adsorption.

Type	Surface Area (m^2^/g)	Average Pore Size (nm)	Total Pore Volume (cm^3^/g)
Raw activated carbon	667.062	1.993	0.332
After conventional adsorption	673.766	1.995	0.336
After ultrasonic adsorption	756.420	1.989	0.376

**Table 10:** Particle size distribution of the activated carbon after conventional and ultrasound-assisted adsorption.

**Adsorption Method**	**Particle Size Distribution (%)**				
	**1700~1180**	**1180~880**	**880~830**	**830~425**	**<425**
Conventional adsorption	95.5	4.4	0.1		
Ultrasonic adsorption	87.2	9.6	1.1	1.5	0.6

## Data Availability

All relevant data supporting the key findings of this study are available within the article or from the corresponding author upon reasonable request.

## References

[1] Xia J.S., Mahandra H., Ghahreman A. (2021). Efficient Gold Recovery from Cyanide Solution Using Magnetic Activated Carbon. ACS Appl. Mater. Interfaces.

[2] Sitando O., Senanayake G., Dai X., Breuer P. (2019). The adsorption of gold(I) on minerals and activated carbon (preg-robbing) in non-ammoniacal thiosulfate solutions—Effect of calcium thiosulfate, silver(I), copper(I) and polythionate ions. Hydrometallurgy.

[3] Figueroa Martinez G.V., Parga Torres J.R., Valenzuela García J.L., Tiburcio Munive G.C., González Zamarripa G. (2012). Kinetic Aspects of Gold and Silver Recovery in Cementation with Zinc Power and Electrocoagulation Iron Process. Adv. Chem. Eng. Sci..

[4] Gámez S., Garcés K., Torre E., Guevara A. (2019). Precious metals recovery from waste printed circuit boards using thiosulfate leaching and ion exchange resin. Hydrometallurgy.

[5] Xu W., Zhou S., Wang B., Zhang P., Tang K. (2022). Efficient adsorption of Au(III) from acidic solution by a novel N, S-containing metal–organic framework. Sep. Purif. Technol..

[6] Yang L., Jia F., Yang B., Song S. (2017). Efficient adsorption of Au(CN${)}_{2}^{-}$ from gold cyanidation with graphene oxide-polyethylenimine hydrogel as adsorbent. Results Phys..

[7] Wang C., Lin G., Zhao J., Wang S., Zhang L. (2020). Enhancing Au(III) adsorption capacity and selectivity via engineering MOF with mercapto-1,3,4-thiadiazole. Chem. Eng. J..

[8] Wang F., Xu W., Zhang K., Tang K. (2022). Efficient and selective adsorption of Au(III) and Pd(II) by trimesoyl chloride-crosslinked polyethyleneimine. React. Funct. Polym..

[9] Wang C.W., Chen S.L., Chen Y.L., Zi F.T., Hu X.Z., Qin X.C., Zhang Y., Yang P., He Y., He P.Q. (2020). Modification of activated carbon by chemical vapour deposition through thermal decomposition of thiourea for enhanced adsorption of gold thiosulfate complex. Sep. Purif. Technol..

[10] Ramírez-Muñiz K., Song S., Berber-Mendoza S., Tong S. (2010). Adsorption of the complex ion Au(CN${)}_{2}^{-}$ onto sulfur-impregnated activated carbon in aqueous solutions. J. Colloid Interface Sci..

[11] Zhang W., Liang Y., Wang J., Zhang Y., Gao Z., Yang Y., Yang K. (2019). Ultrasound-assisted adsorption of Congo red from aqueous solution using MgAlCO_3_ layered double hydroxide. Appl. Clay Sci..

[12] Daware G.B., Gogate P.R. (2021). Removal of pyridine using ultrasound assisted and conventional batch adsorption based on tea waste residue as biosorbent. Environ. Technol. Innov..

[13] Jing G., Zhou Z., Song L., Dong M. (2011). Ultrasound enhanced adsorption and desorption of chromium (VI) on activated carbon and polymeric resin. Desalination.

[14] Gao X., Liu J., Li M., Guo C., Long H., Zhang Y., Xin L. (2020). Mechanistic study of selective adsorption and reduction of Au (III) to gold nanoparticles by ion-imprinted porous alginate microspheres. Chem. Eng. J..

[15] Wang J., Guo X. (2022). Rethinking of the intraparticle diffusion adsorption kinetics model: Interpretation, solving methods and applications. Chemosphere.

[16] Groszek A.J., Partyka S., Cot D. (1991). Heats of adsorption of gold chloride and cyanide complexes from aqueous solutions on graphitized carbon black and a coconut active carbon. Carbon.

[17] Gahrouei A.E., Rezapour A., Pirooz M., Pourebrahimi S. (2024). From classic to cutting-edge solutions: A comprehensive review of materials and methods for heavy metal removal from water environments. Desalin. Water Treat..

[18] Lin G., Hu T., Wang S., Xie T., Zhang L., Cheng S., Fu L., Xiong C. (2019). Selective removal behavior and mechanism of trace Hg(II) using modified corn husk leaves. Chemosphere.

[19] Feng B., Yao C., Chen S., Luo R., Liu S., Tong S. (2018). Highly efficient and selective recovery of Au(III) from a complex system by molybdenum disulfide nanoflakes. Chem. Eng. J..

[20] Saman N., Rashid M.U., Lye J.W.P., Mat H. (2018). Recovery of Au(III) from an aqueous solution by aminopropyltriethoxysilane-functionalized lignocellulosic based adsorbents. React. Funct. Polym..

[21] Sedira N., Bouranene S., Gheid A. (2022). Ultrasound-assisted adsorption of Pb ions by carbonized/activated date stones from singles/mixed aqueous solutions. J. Indian Chem. Soc..

[22] Adebisi G.A., Chowdhury Z.Z., Alaba P.A. (2017). Equilibrium, kinetic, and thermodynamic studies of lead ion and zinc ion adsorption from aqueous solution onto activated carbon prepared from palm oil mill effluent. J. Clean. Prod..

[23] Egbosiuba T.C., Abdulkareem A.S., Kovo A.S., Afolabi E.A., Tijani J.O., Auta M., Roos W.D. (2020). Ultrasonic enhanced adsorption of methylene blue onto the optimized surface area of activated carbon: Adsorption isotherm, kinetics and thermodynamics. Chem. Eng. Res. Des..

[24] Niu H., Volesky B. (2001). Gold adsorption from cyanide solution by chitinous materials. J. Chem. Technol. Biotechnol..

[25] Alaei R., Javanshir S., Behnamfard A. (2020). Treatment of gold ore cyanidation wastewater by adsorption onto a Hydrotalcite-type anionic clay as a novel adsorbent. J. Environ. Health Sci. Eng..

[26] Swantomo D., Faturrahman I.R., Basuki K.T., Wongsawaeng D. (2020). Chitosan-polyacrylamide graft copolymers prepared with gamma irradiation for gold cyanide adsorption. Polym.-Plast. Technol. Mater..

[27] Ok Y.S., Jeon C. (2014). Selective adsorption of the gold–cyanide complex from waste rinse water using Dowex 21K XLT resin. J. Ind. Eng. Chem..

[28] Xia J., Marthi R., Twinney J., Ghahreman A. (2022). A review on adsorption mechanism of gold cyanide complex onto activation carbon. J. Ind. Eng. Chem..

[29] Belwal T., Li L., Yanqun X., Cravotto G., Luo Z. (2020). Ultrasonic-assisted modifications of macroporous resin to improve anthocyanin purification from a *Pyrus communis* var. Starkrimson extract. Ultrason. Sonochem..

[30] Yu Q., Fan L., Li J. (2020). A novel process for asparagus polyphenols utilization by ultrasound assisted adsorption and desorption using resins. Ultrason. Sonochem..

